# A Laboratory Experimental Study: An FBG-PVC Tube Integrated Device for Monitoring the Slip Surface of Landslides

**DOI:** 10.3390/s17112486

**Published:** 2017-10-30

**Authors:** Kai Wang, Shaojie Zhang, Jiang Chen, Pengxiao Teng, Fangqiang Wei, Qiao Chen

**Affiliations:** 1 Key Laboratory of Mountain Hazards and Earth Surface Process, Institute of Mountain Hazards and Environment, Chinese Academy of Sciences, Chengdu 610041, China; qianlimingyu@163.com; 2 University of Chinese Academy of Sciences, Beijing 100049, China; 3 College of Architecture & Environment, Sichuan University, Chengdu 610065, China; chxifei@126.com; 4 Institute of Acoustics, Chinese Academy of Sciences, Beijing 100190, China; px.teng@mail.ioa.ac.cn; 5 Chongqing Institute of green and Intelligent Technology, Chinese Academy of Sciences, Chongqing 400714, China; fqwei@imde.ac.cn (F.W.); chenqiao@cigit.ac.cn (Q.C.)

**Keywords:** FBG, PVC tube, slip surface monitoring, slope model

## Abstract

A new detection device was designed by integrating fiber Bragg grating (FBG) and polyvinyl chloride (PVC) tube in order to monitor the slip surface of a landslide. Using this new FBG-based device, a corresponding slope model with a pre-set slip surface was designed, and seven tests with different soil properties were carried out in laboratory conditions. The FBG sensing fibers were fixed on the PVC tube to measure strain distributions of PVC tube at different elevation. Test results indicated that the PVC tube could keep deformation compatible with soil mass. The new device was able to monitor slip surface location before sliding occurrence, and the location of monitored slip surface was about 1–2 cm above the pre-set slip surface, which basically agreed with presupposition results. The monitoring results are expected to be used to pre-estimate landslide volume and provide a beneficial option for evaluating the potential impact of landslides on shipping safety in the Three Gorges area.

## 1. Introduction

Numerous deep-seated landslides with undetermined slip surfaces are widely distributed in the Three Gorges area. These landslides are easily affected by rainstorms or reservoir water level variations and may suddenly slide along slip surfaces, causing catastrophe for local residents [1,2,3,4]. The construction of landslide control works is an effective method for landslide defense. However, there are many landslides in need of disaster mitigation, and the construction of landslide control works will cost a great deal of money and take a long time [5]. Therefore, landslide monitoring in the Three Gorges area is essential for disaster mitigation [6,7]. Currently, the main monitoring purpose within this area is determining whether a landslide occurs or not by analyzing the real-time monitoring data [8,9,10,11,12]. Monitoring modes that only have an early warning function cannot fundamentally prevent landslide occurrence. When a deep-seated landslide cannot be stopped from sliding into the reservoir, besides evacuating residents with the help of the monitoring data, the remaining important mitigation tasks in this area are to assess the possibility of blocking river channel and the potential impact of the surge on shipping safety. These dangerous situations closely relate to the landslide volume sliding into the reservoir. Apparently, current monitoring methods using only landslide occurrence or non-occurrence is not adequate for landslides mitigation within the Three Gorges area, and accordingly it is necessary to estimate the potential landslide volume in advance of landslide occurrence. With respect to the pre-estimation of landslide volume, slip surface monitoring is expected to be a useful option because the monitoring result could be used to generate a slip surface profile, and landslide volume would be pre-estimated using the slip surface profile as an integration domain [13]. A monitoring device with the ability to monitor the slip surface will be helpful to provide more mitigation information and serve for improved mitigation work in the Three Gorges area.

Fiber optical sensors (FOS) have the advantages of anti-corrosion, being waterproof, tiny size, high sensitivity, immunity to electromagnetic interference and long-term stability, so they have been widely used to monitor structural health [14,15] and geological hazards [16,17,18]. For slip surface monitoring using the FOS technique, two typical FOS-based modes including the internal displacement monitoring method [19,20] and strain monitoring method [21] were adopted. Slope instability is considered to be closely related to slope deformation [22]. Accordingly, FBG-based inclinometers were designed to measure internal displacement and locate the slip surface by analyzing displacement data [23,24]. However, the detectable displacement is the result of strain accumulation within the soil mass. That is to say, the stress state in soil mass has changed prior to the displacement deformation. In this view, the displacement monitoring results would lag behind the real evolution of the stress state in a landslide. It is known that strain monitoring is a more direct and sensitive measurement to reflect the mechanical mechanism of a landslide compared to displacement monitoring [25]. Some scholars have attempted to monitor the slip surface according to strain distribution in slope mass or geotechnical structures [26,27,28,29,30]. Generally, the installation modes of fiber sensors could be divided into two categories: (1) Fiber sensors are directly embedded in soil mass with no carrier. For example, fibers coated by heat shrinkage tube were embedded in a slope model to measure horizontal strain distribution at different elevations using Brillouin Optical Time Domain Analysis (BOTDA) technique [21], and the slip surface could be located by identifying the maximum strain in each layer. Additionally, a slope failure model was tested using FBG sensors, in which FBG sensors were respectively embedded at three soft interlayers. Based on the time–strain relationships as well as the slide displacement through monitoring the slope strain, the weak structural plane can be located [28]. Because of granular material properties of soil mass, the above two typical monitoring methods cannot ensure that the sensing fibers effectively fix on the soil mass in practice. This situation may indicate a phenomenon of relative sliding between fiber sensors and soil layers when an external force acts on these testing models, which are supposed to be even more serious when the loose or soft soil mass used in testing models. Consequently, this difficulty (relative sliding) in guaranteeing compatible deformation of soil mass and fiber will weaken the monitoring reliability; (2) Design an integration device by fixing fiber sensors on a specific carrier. For example, Pei et al. (2013) [26] and Zhu et al. (2015) [27] fixed FBG strain sensors to a soil nail and designed an FBG-based device to monitor landslides. The main characteristics of these integration devices are layer arranged in testing slope models. According to their research, the maximum strain of the soil nail at each soil layer represented the passing point of the potential slip surface, and then the whole slip surface could be identified by connecting each passing point. Likewise, Sun et al. (2017) [29] installed FBG sensors on the geogrid and embedded these detecting devices at two layers within a slope model. The strain of the geogrid was applied to evaluate the slope model stability. Indeed, the carriers could improve the compatible deformation of the soil and sensing fibers, but the fiber sensors still need to be layer embedded into the soil mass during the slope model construction. It should be noted that the scheme of embedding fiber sensors into soil mass is practical in laboratory conditions, because the slope model is not too large. However, for a natural landslide, the layer embedded method means that soil mass need to be excavated layer by layer in order to install fiber sensors and their corresponding carrier. Another problem is that most natural landslides have huge volume with undiscovered slip surfaces. This situation is extremely unfavorable for field construction, because the layer embedded method needs huge earthworks and a large quantity of sensing fibers.

This paper addresses the problems in FBG- or BOTDA-based slip surface monitoring methods at present, and a monitoring device for the slip surface integrating FBG and PVC tube is proposed. A series of slope mode tests were conducted in laboratory conditions to verify its reliability. The potential application of this new device was also discussed based on the slip surface monitoring results. This new device could be applied in natural landslide monitoring with the help of borehole technology and can be expected to provide a scientific basis for slip surface monitoring using the FBG technique.

## 2. Monitoring Device Design

**Figure 1:**
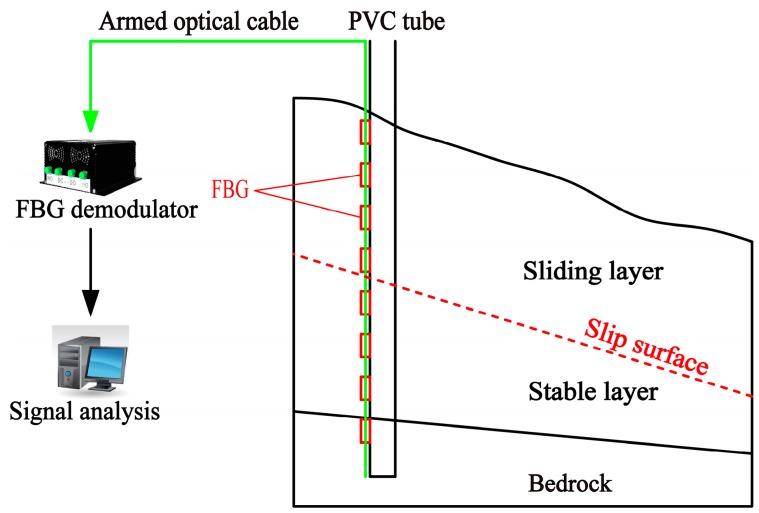
Sketch of FBG-PVC integration device for measuring sliding surface position.

The proposed monitoring device includes a sensing system and a force-light conversion system (Figure 1).The former, based on the strain sensing technique, is composed of the FBG demodulator, FBG strain sensors and signal transmission fiber optic cable, and the latter mainly of the PVC tube and slope mass. Compared with steel tube or other materials with high stiffness [31], PVC tube has a lower elastic modulus that allows it to keep deformation compatible with soil mass as far as possible. Therefore, PVC tube was chosen as the carrier for FBG sensors. The quasi-distributed FBG sensors are installed on the PVC tube at different elevations instead of being layer embedded in soil mass (Figure 1), so the quantity demand for sensing fibers would be reduced without excavating a huge soil mass. 

## 3. Monitoring Theory

### 3.1. Monitoring Principle of FBG

The core material of the optic fiber is characterized by photosensitivity, and when illuminated by ultraviolet light its refractive index varies with changing light intensity. This behavior allows the UV write-in technique to be adopted to write the coherent field of incident light into the fiber core so as to have periodic variations of the refractive index in the core area, where the light of a specific wavelength can be reflected. The English physicist Bragg found the reflection law of FBG:

*λ_B_ = 2nΛ* (1)

where *λ_B_* is the reflected light wavelength; *n* is the effective refractive index; *Λ* is the optical grating’s period.

The reflected light wavelength *λ_B_* of FBG varies with the change of temperature and strain, so its wavelength shift *∆λ_B_* versus the increment of strain *∆ε* and of temperature *∆T* can be expressed as:

*∆λ_B_* = *K_ε_∆ε* + *K_T_∆T* (2)

where *K_ε_* and *K_T_* denote the strain sensibility coefficient (nm/µε) and the temperature sensibility coefficient (nm/°C), respectively [14,18]. It needs to be noted that all the tests have been completed under normal temperature level, and time for data collection is very short. It is therefore reasonable to assume that the shift of central wavelength is irrelevant to temperature variation, so *∆λ_B_* in the expression (2) is relevant only to the strain increment *∆ε*. Accordingly, the expression (2) could be transformed as:

*∆ε* = *∆λ_B_/K_ε_* (3)

The bragger wavelength shift can be converted into strain value using the expression (3), where the strain sensibility coefficient *K_ε_* = 1.2 [18].

### 3.2. Relationship between PVC Tube and Slip Surface Location

**Figure 2:**
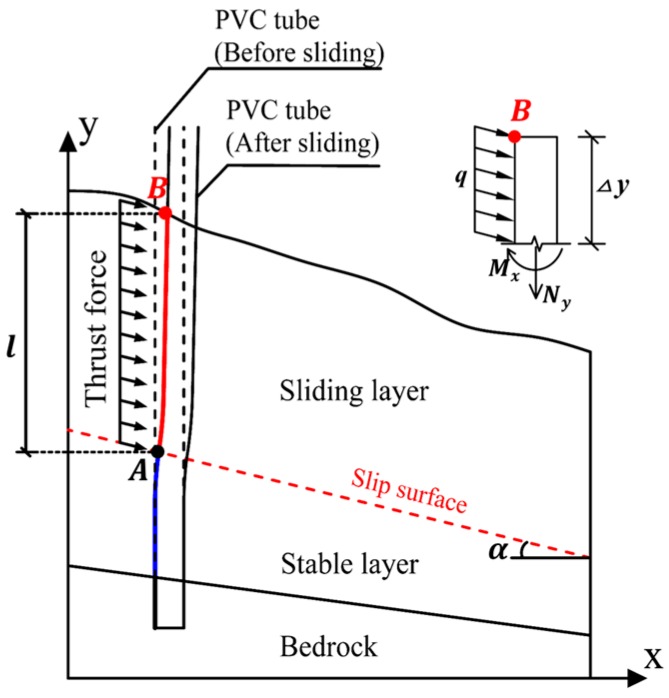
Schematic diagram of relationship between PVC pipe and sliding surface.

As shown in Figure 2, the left side of the PVC tube above the sliding surface (red line in Figure 2) was in compression state and accordingly yielded compressive strain, when an external force was applied. The thrust force listed in Figure 2 caused that the tube had a tendency of pulling out from soil mass. However, this tendency was constrained and stopped, because the tube end was fixed in the bed rock. Just because this tendency was limited, the left side of the PVC tube beneath the slip surface was in tensile state and accordingly yielded tensile strain. Apparently, slip surface could be considered the interface of compressive and tensile zones of PVC tube. Here the sign “+” was used to represent the tensile strain and sign “−” signify compressive strain. So the intersection point of tube and the slip surface can be considered the demarcation point, where the strain sign would change. The key issue for slip surface monitoring lies in identifying the demarcation point of the compressive and tensile zone, e.g., the point *A* (marked by black circle) in the Figure 2. The demarcation point *A* could be identified by the strain distribution curve of PVC tube according to strain data measured by quasi-distributed FBG sensing arrays.

The end of PVC tube was fixed in the bedrock without any rotation. The effect of the boundary conditions to the strain measurement could be interpreted using the Euler-Bernoulli beam theory [32,33]. The mechanical mode of PVC tube in the sliding layer is equivalent to an elastic axisymmetric beam subject to transverse loading and/or axial loading. The thrust force transmitted from soil mass is simplified as uniform loading with intensity *q*. The distribution of strain *ε* relates to the distribution of axial force *N_y_* and bending moment *M_x_*, which can be expressed by

(4) $$\varepsilon =\varepsilon_{y}+\varepsilon_{x}=\frac{N_{y}}{\mathrm{EA}}+\frac{M_{x}}{{\mathrm{EI}}_{x}}R$$

while *N_y_* and *M_x_* could be calculated by

*N_y_* = *q∆ysinα* (*∆y* = *0–l*) (5)

*M_x_* = *½q∆y^2^cosα* (*∆y = 0–l*) (6)

where *ε_y_* is axial strains induced by axial loading *N_y_*; *ε_x_* is strain due to transverse loading in the *x* direction, which is related to *M_x_*; *R* is the radius of PVC tube; *α* is the inclination angle of slip surface, with a range of [0°,90°]; *E*, *A*, *I_x_* is elastic modulus, the cross sectional area, moment of inertia with respect to *x* axis of PVC tube, respectively; *l* is length of PVC tube subject to thrust force. 

Using the cross-section method in the mechanics of materials [34,35], the PVC tube is made up of infinite cross sections. Extracting a cross section of the PVC tube in the sliding layer, *∆y* is the distance from the point B to the cross section(Figure 2), while *∆y* = 0 at point B, and *∆y* = *l* at point A. According to the expressions (5) and (6), with the rising of *∆y* from point B to point A, the *N_y_* and *M_x_* on the cross sections will increase and the maximum *N_y_* and *M_x_* will appear at the cross sections of PVC tube near the slip surface. Substituting the expressions (5) and (6) into (4), the strain would also show a rising trend from point B to point A, and the maximum strain occurs near the slip surface.

## 4. Slope Model Test

Based on the relationship of the slip surface location and the strain distribution rule on the PVC tube, a slope model with a pre-set slip surface was designed, seven working conditions with different soil properties were constructed in laboratory. The main purpose of the model tests were to identify the location of slip surface by analyzing the variation characteristics of strain at each monitoring point on the tube, and to compare the identified results with the location of the horizontal pre-set slip surface in order to verify the reliability of the FBG-based device.

### 4.1. Properties of PVC Tube and FBGs

The geometric size and physical properties of PVC tube is shown in Table 1. In the condition of a same wall thickness, a larger diameter means a larger inertia moment and cross sectional area. Equation 4 indicates that the PVC tube with larger inertia moment will tend to keep an original state with a smaller deformation. So it follows that the diameter of the PVC tube can significantly influence the strain measurement result. The length of each FBG sensor is 1 cm. The interrogator has four channels with a product model ID FS2200. The four available optical channels have simultaneous acquisition meaning that the sensing network can have a large number of FBGs interrogated at the same time. Its maximum range of wavelength measurement is 100 nm (1500 to 1600 nm) with the resolution of 5 pm, and its maximum sample rate is 100 Hz.

### 4.2. Installation of FBGs

**Figure 3:**
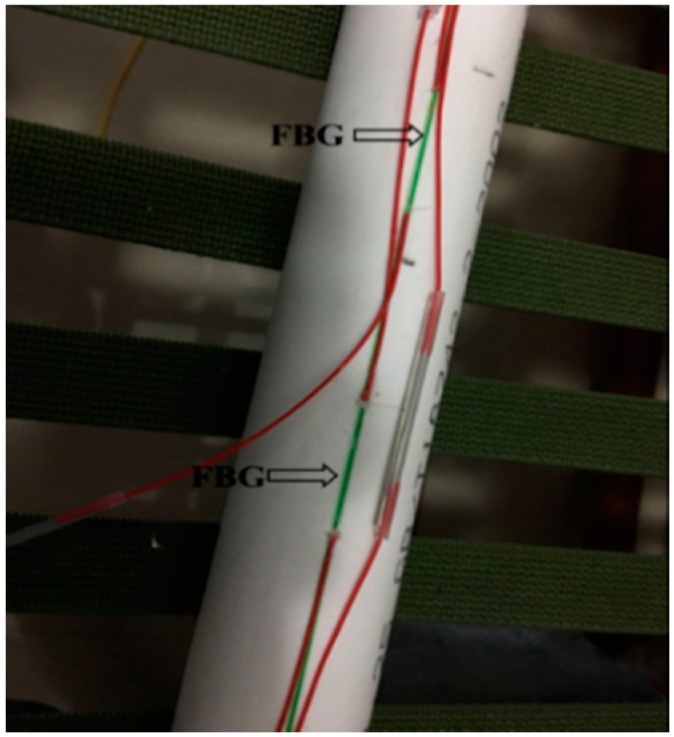
Mounting of FBGs.

**Figure 4:**
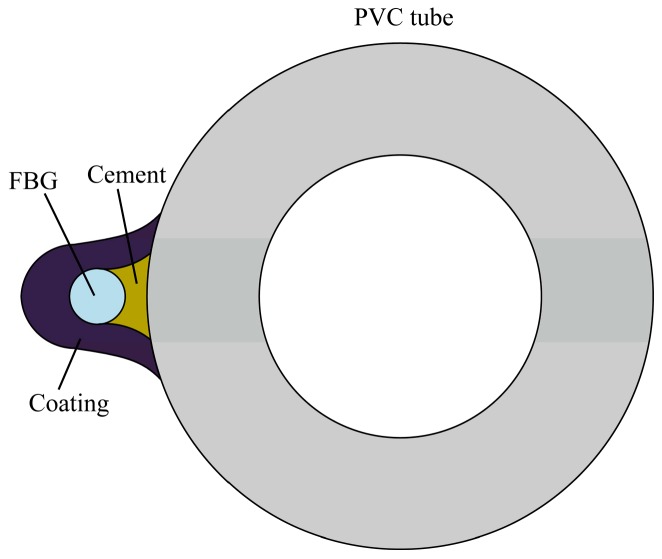
Cross section of FBG installation.

The single-mode bare FBG fibers were chosen as strain sensing elements. The installation processes were as follows: (1) the bare FBG fibers were fixed on the outer surface of a PVC tube using quick-dry cyanoacrylate adhesive (Figure 3), the PVC tube has a length of 200 cm(due to the space limit in the laboratory condition) and a radius of 3 cm; (2) a heat shrinkage tube with 3 mm diameter was cut open and packaged on the FBG sensors to form a protecting coating (Figure 4); (3) glass cement was used to seal any gaps between FBG sensors and the protecting coating.

### 4.3. Slope Model

**Figure 5:**
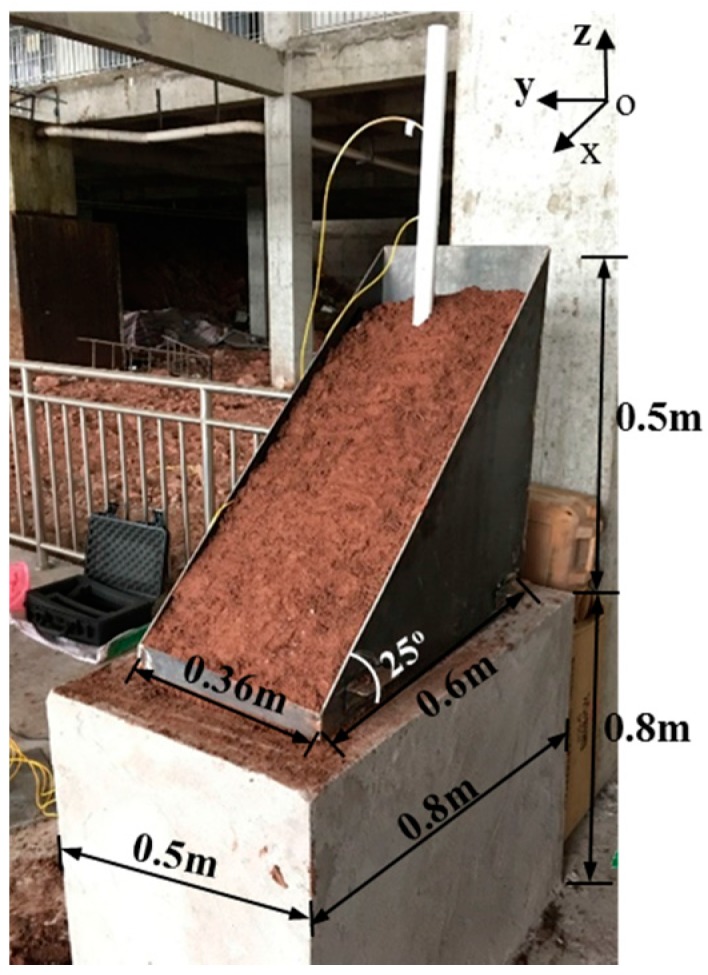
Slope model. (Z axis is perpendicular to the “xoy” plane).

**Figure 6:**
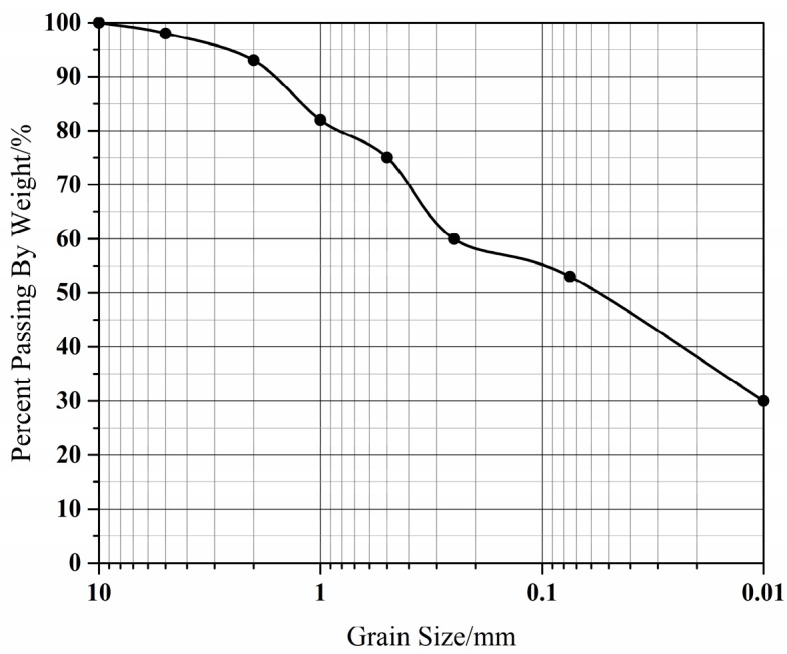
Particle size distribution of test soil.

The experimental model consisted of a cement tank and a sliding mass chamber (Figure 5); the former part was totally fixed on the ground in order to simulate the bedrock and stable layer, the latter was made up of detachable steel plates and used to simulate the sliding mass. Soil used in the tests was silt clay, which was widely distributed in the Three Gorges reservoir region, China. The particle size distribution was shown in Figure 6, the fine particle content (<0.1 mm) exceeded 50%. Compaction tests in the laboratory indicated that the maximum dry density was 1.89 g/cm^3^ and its optimum water content was 19%.

**Figure 7:**
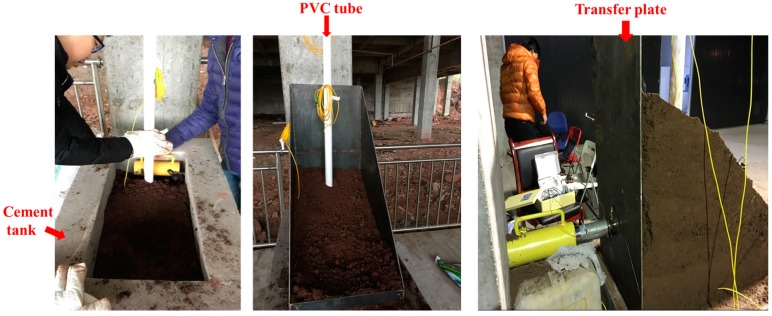
Construction process of slope model.

The filling processes of the slope model were as follows(Figure 7): (1) the detecting device by integration of PVC tube and FBG sensors was fixed at the bottom of the cement tank; (2) the soil sample was filled and tamped in the cement tank until the tank was full, then the sliding mass chamber was put on the cement tank and also filled and tamped using the same soil sample; (3) removed sliding mass chamber and formed a sliding mass with an angle of 25° (Figure 5).

**Figure 8:**
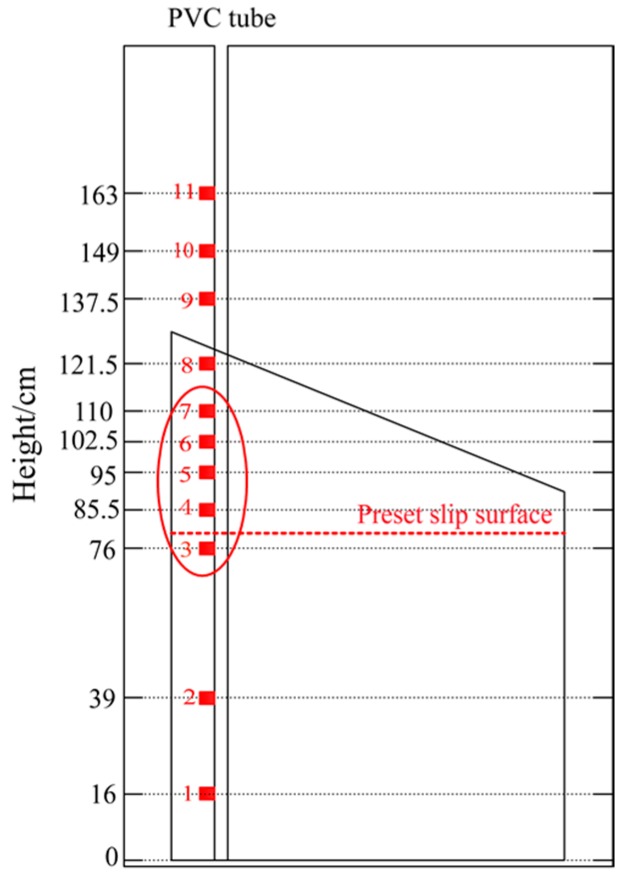
Monitoring points arrangement.

Because the soil contained in the cement tank and the sliding mass chamber was tamped respectively, a horizontal “construction joint” was formed at the interface of cement tank and the sliding mass chamber, which was similar to the weak structure plane within slope mass. Slope mass tended to slide along this interface attributing to the horizontal thrust force and constrain of steel plate in Figure 8, therefore the interface was taken as the pre-set slip surface. During each test, soil mass above the slip surface would move along the horizontal pre-set slip surface. Soil mass beneath the slip surface was considered the stable layer, because the cement tank was totally fixed on the ground. 

### 4.4. Arrangement of FBG Sensors

The preset slip surface is a key component within our testing model in order to verify the effectiveness of the proposed device. Therefore, a higher monitoring precision was necessary nearby the pre-set slip surface meaning a smaller arrangement space of FBG sensors around this pre-set surface. As shown in Figure 8, eleven FBG strain sensors were installed on the left side of the PVC tube with unequal intervals. Three sensors (numbered by 1, 2, and 3) were arranged below the slip surface in order to monitor the strain state of the PVC tube embedded in the stable layer (cement tank), while the others were above the slip surface. The sliding mass was subject to the thrust force and moved along the interface at the *z* direction in Figure 5, in which five FBG sensors were arranged in order to monitor the strain state of this embedded tube section. FBG strain sensors such as sensors 3–7 in Figure 8 were intensively installed nearby the pre-set slip surface for the purpose of a higher monitoring precision. The rest three sensors were installed at the free end of PVC tube for comparing to the monitoring results of other points. Among the eleven monitoring points, sensors 3 and 4 were the nearest points to the pre-set slip surface. The strain sign (“+” or “−”) would be most likely to change between those two points.

### 4.5. Experiment Scheme

A hydraulic jack and a transfer plate (Figure 7) with a thickness of 10 mm were chosen as the loading system. Because the stiffness of transfer plate is very large and the actual loading is relatively small, it is reasonable to neglect the local deformation of transfer plate during the loading process. Therefore, the transfer plate is able to uniformly transfer the load to the soil mass. 

The progressive sliding force was simulated by the hydraulic jack in the form of gradual loading mode, and then an even surface force was applied at the back of slope model attributing to the transfer plate between the slope model and the hydraulic jack. The loading stopped at the moment of the sliding mass beginning to slide along the pre-set slip surface. As listed in Table 2, seven cases were designed for investigating the experimental results under different physical state of test soil. The soil sample in case 3 had the maximum compaction degree and the corresponding soil water content was 19%, in contrast, the soil in case 6 had the lowest compaction degree. 

## 5. Test Result Analysis

### 5.1. Strain Monitoring Result of FBG Sensing Array

**Figure 9:**
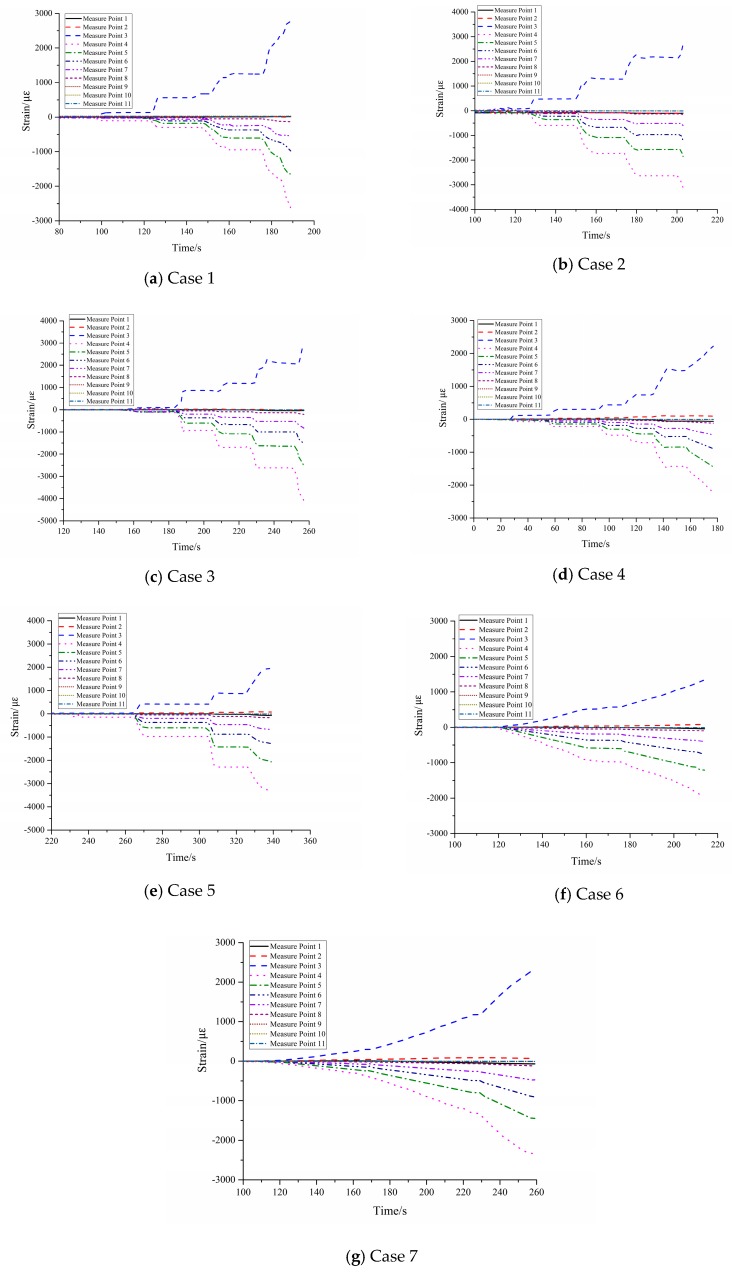
Time history curves of strain in cases 1–7 (Horizontal coordinate axis: the loading time; Vertical coordinate axis: the strain of measure points).

The time history curves of all the cases (1–7) shown in Figure 9 indicated that all monitoring points in sliding mass were in compressive state and yielded the compressive strains, whereas the monitoring points beneath the pre-set slip surface yielded the tensile strains. Accordingly, the strain sign totally changed from monitoring point 3 to 4. Test results made it plausible to take the slip surface as the interface of compression and tensile zone of PVC tube. 

The compatible deformation of PVC tube and soil mass was the premise to ensure the reliability of monitoring results, which could be examined through the strain distribution of PVC tube near the pre-set slip surface during tests. As a gradually increasing surface force was applied to the slope model, the slope mass had a sliding tendency along the pre-set slip surface (the weak structure plane), and the strain accumulation in the slope mainly concentrated in the soil closing to the pre-set slip surface. This situation indicated that there were supposed to be maximum strain accumulations at monitoring points 3 or 4 on the tube, because they were the closest points to the pre-set slip surface. 

As shown in Table 3, the maximum compressive and tensile strain were respectively at point 3 and 4, meaning that the strain accumulation on the PVC tube was basically in accordance with the soil mass. Therefore, the capacity of PVC tube to keep deformation compatible with soil mass was reliable.

### 5.2. Slip Surface Monitoring Result Analysis

**Figure 10:**
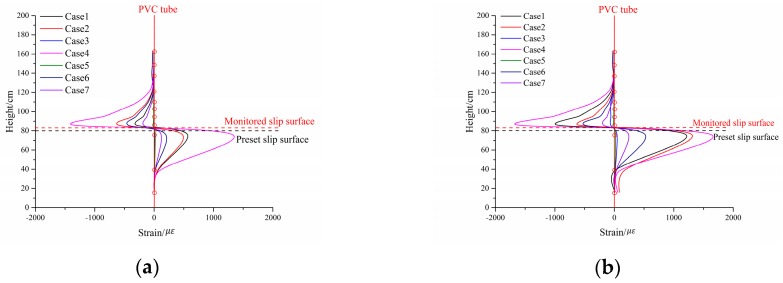

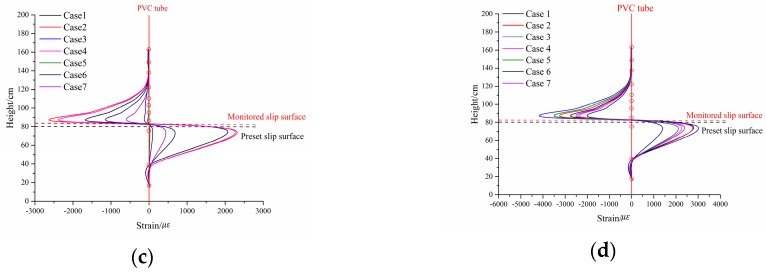
Strain curves under different loading moments (red solid lines represent PVC tube, red circles represent the monitoring points location). (**a**) Loading time: 140 s; (**b**) Loading time: 160 s; (**c**) Loading time: 180 s; (**d**) Final loading moment.

Monitoring slip surfaces can reliably locate the potential slip surfaces before landslide occurrence and provide more detailed warning information to residents. Therefore, an effective slip surface monitoring device should be able to locate the slip surface at different loading times before slide occurrence [25]. The strain distribution curves fitted through Bezier splines using the discrete strain data are shown in Figure 10, which would be helpful to examine the effectiveness of slip surface monitoring result. As shown in Figure 10, the strain distribution rules on the monitoring device in all cases were basically consistent, which can be drawn as follows: the tube can be divided into compressive and tensile zones if taking the tube height of about 81–82 cm as the boundary point. Above this boundary point, the tube was in compressive state and the compression strain gradually increased as the tube height decreased. However, this rule of compressive strain ceased abruptly as the tube height dropped below the boundary point immediately following the maximum level of tensile strain, which indicated the tensile state dominating in this zone bellowing the boundary point. Based on the theoretical analysis in Section 3.2, the point with about 81 cm or 82 cm was identified as the sliding location and is marked by red dotted lines in Figure 10. As shown in Figure 10, the monitored slip surface position is about 1 cm higher than the pre-set slip surface. 

## 6. Conclusions

Based on the FBG strain sensing technique, a new FBG-PVC tube integrated device was proposed to monitor the slip surface in a landslide. The characteristics of the new device mainly lie in the compatible deformation of the soil mass and the PVC tube and different arrangement space of FBG sensors on the PVC tube. A slope model with a pre-set slip surface was designed, and seven cases with different soil properties were investigated to verify the reliability of the new device. Some conclusions are drawn as follows:

(1) The new device not only takes full advantage of the high sensitivity of FBG strain sensors, but also makes full use of the capacity of PVC tube to keep deformation compatible with soil mass. The installation mode of the proposed monitoring device in soil mass indicates that the sensor quantity is expected to be lower than the layer-embedded method. Overall, the new device is cost-effective and convenient for field construction without excavating massive soil mass. 

(2) The location of strain sign variation (from “−” to “+”) is considered to be the slip surface. Following this rule, the testing results showed that the identified slip surface is only 1–2 cm above the pre-set slip surface (or real sliding surface), and a monitoring error of about 1.25–2.5% is acceptable. The monitoring method for locating slip surface is therefore reliable.

It should be noted that a key issue arising from the laboratory conditions is the scale factor effect. Full-scale field tests are crucial to improve the understanding of the strain behavior of PVC tube near the slip surface. Therefore, further studies will be conducted to adopt this device to monitor the slip surface within an actual landslide and verify the effectiveness of the proposed monitoring device in the field.

## Figures and Tables

**Table 1:** Properties of PVC tube.

Outer Diameter D/cm	Inner Diameter d/cm	Wall Thickness t/cm	Radius R/cm	Length L/cm	Inertia Moment *I*/(10 × mm^4^)
6.0	3.0	1.5	3.0	200.0	59.7

**Table 2:** Experiment schemes.

Case	Dry Density	Compaction Degree	Water Content (%)
1	1.68	89%	15
2	1.75	93%	16
3	1.82	96%	19
4	1.58	84%	18
5	1.78	94%	18
6	1.49	79%	22
7	1.63	86%	13

**Table 3:** Measuring results of maximum strain in cases 1–7.

Cases	Maximum Compressive Strain		Maximum Tensile Strain		Loading Time/s	Maximum Loading Force/kN
	Value/*με*	Location	Value/*με*	Location		
1	2644.05	4	2753.80	3	188	3.05
2	3113.72	4	2694.19	3	202	3.48
3	3996.87	4	2931.82	3	257	4.57
4	2285.83	4	2220.15	3	178	2.61
5	3357.88	4	2013.24	3	342	3.92
6	1913.45	4	1342.16	3	218	2.18
7	2412.17	4	2343.55	3	260	2.83
